# Supplementary material for: Increased risk of hospitalisation and intensive care admission associated with reported cases of SARS-CoV-2 variants B.1.1.7 and B.1.351 in Norway, December 2020 –May 2021
Source: PLoS One. 2021 Oct 11;16(10):e0258513. doi: 10.1371/journal.pone.0258513 (PMC8504717; doi:10.1371/journal.pone.0258513)
Supplement: S1 File — (DOCX) [file pone.0258513.s001.docx]

Supplementary materials: Increased risk of hospitalisation and intensive care admission associated with infection with SARS-CoV-2 variants B.1.1.7 and B.1.351 in Norway, December 2020 – May 2021

**Table of contents**

[1. ICD-10 and ICPC-2 codes for the identification of cases with underlying comorbidities 1](#_Toc74142784)

[2. Number of cases of COVID-19 with available data on virus variant, by variant category and week of sampling 2](#_Toc74142785)

[3. Additional statistical analysis 2](#_Toc74142786)

[3.1 Assessment of representativeness of study population 2](#_Toc74142787)

[3.2 Sensitivity analyses 4](#_Toc74142788)

[3.3 Results from logistic regression when the variant was included as a 3-level categorical variable 5](#_Toc74142789)

## 1. ICD-10 and ICPC-2 codes for the identification of cases with underlying comorbidities

**Table S1. ICD-10 codes from the Norwegian Patient Registry and ICPC-2 codes from the Norway Control and Payment of Health Reimbursement database used to identify cases with underlying comorbidities.**

| **Underlying comorbidity** | **Specifications** | **ICD10-codes** | **ICPC-2 codes** |
| --- | --- | --- | --- |
| Cardiovascular diseases, not including hypertension |  | I05, I06, I07, I08, I09, I2, I31, I32, I34, I35, I36, I37, I39, I40, I41, I42, I43, I46, I48, I49, I50, I60, I61, I62, I63, I64, I69.1, I69.2, I69.3, I69.4, I69.8, I69.0 | K74, K75, K76, K77, K78, K82, K83, K90, K91 |
| Chronic pulmonary diseases, including asthma |  | J41, J42, J43, J44, J45, J46, J47, J84, J98, E84 | R95, R96 |
| Compromised immune function | Organ transplantation, immune deficiency disorders, autoimmune conditions treated with immunosuppressants | Z94.0, Z94.1, Z94.2, Z94.3, Z94.4, Z94.8, D80, D81, D82, D83, D84, G35, M05, M08, M06, M07, M09, M13, M14, K50, K51 |  |
| Neurological and musculoskeletal disorders with compromised lung or cough function |  | G1, G20, G21, G23, G24, G40.5, G61.0, 70, G71, G80.0, G80.2, G80.3, F72, F73, F84.0, F84.1, Q05.0, Q05.1, Q05.2, Q05.3, Q05.04, Q05.5, Q05.6 |  |
| Diabetes |  | E10, E11, E12, E13, E14 | T89, T90 |
| Active cancer treatment or hematological cancer |  | C81, C82, C83, C84, C85, C86, C87, C88, C89, C90, C91, C92, C93, C94, C95, C96, D45, D45, D47, C0, C1, C2, C3, C4, C5, C6, C7, C80, D32, D33, D35.2, D35.3, D35.4, D42, D43, D44.2, D44.3, D44.4 |  |
| Other risk groups | Dementia, chronic kidney and liver disease, obesity | N18.3, N18.4, N18.5, K70.4, K72, F00, F01, F02, F03, G30, G31, E66 | P70, T82 |

## 2. Number of cases of COVID-19 with available data on virus variant, by variant category and week of sampling

In total, 65,040 cases of COVID-19 were reported in the study period, of which 32,163 had data on virus variant (49%) (table S2). This includes cases who were vaccinated, did not have a national identity number and were admitted to hospital with another main cause of hospitalisation than COVID-19, or unknown main cause. These cases were removed from the analysis dataset, which was based on 60,606 cases of which 29,979 (49%) had known variant data.

**Table S2. Number of cases of COVID-19 with available data on virus variant, by variant category and week of sampling, Norway, 28 December 2020 – 2 May 2021**

| **Week of sampling** |  | **B.1.1.7** | | **B.1.351** | | **Non-VOC** | | **Other VOC*** | | **Unclassified**** | |
| --- | --- | --- | --- | --- | --- | --- | --- | --- | --- | --- | --- |
|  | **Total** | **n** | **%** | **n** | **%** | **n** | **%** | **n** | **%** | **n** | **%** |
| Week 53-1 | 680 | 90 | 13 | 2 | 0.3 | 587 | 86 | 0 | 0 | 1 | 0.2 |
| Week 2-3 | 898 | 134 | 15 | 1 | 0.1 | 760 | 85 | 0 | 0 | 3 | 0.3 |
| Week 4-5 | 1,817 | 491 | 27 | 34 | 1.9 | 1,274 | 70 | 0 | 0 | 18 | 1.0 |
| Week 6-7 | 2,427 | 1,306 | 54 | 89 | 3.7 | 978 | 40 | 0 | 0 | 54 | 2.2 |
| Week 8-9 | 5,631 | 4,227 | 75 | 183 | 3.3 | 935 | 17 | 1 | 0.02 | 285 | 5.1 |
| Week 10-11 | 5,742 | 4,979 | 87 | 128 | 2.2 | 217 | 3.8 | 2 | 0.04 | 416 | 7.2 |
| Week 12-13 | 5,053 | 4,500 | 89 | 94 | 1.9 | 85 | 1.7 | 1 | 0.02 | 373 | 7.4 |
| Week 14-15 | 5,466 | 4,938 | 91 | 65 | 1.2 | 64 | 1.2 | 3 | 0.03 | 396 | 7.2 |
| Week 16-17 | 4,449 | 4,171 | 94 | 9 | 0.2 | 48 | 1.1 | 13 | 0.3 | 208 | 4.7 |
| Whole period | 32,163 | 24,836 | 77 | 605 | 1.9 | 4,948 | 15 | 20 | 0.06 | 1,754 | 5.5 |

* Includes 7 P.2 and 13 B.1.617.2.

** Unclassified cases include samples where B.1.1.7, B.1.351, other VOC and non-VOC could not clearly be distinguished.

## 3. Additional statistical analysis

### 3.1 Assessment of representativeness of study population

We assessed the representativeness of our study population by comparing the characteristics of cases in our study cohort (B.1.1.67, B.1.351 and non-VOC) and notified cases. We found differences between our study cohort and notified cases with regards to county of residence, sampling week, age, number of comorbidities, and hospitalisation (table S3). Differences in county and sampling week reflect the evolution of the outbreak as well as the introduction of PCR screening methodology for virus variants at the primary diagnostic laboratories. The proportion of cases in our study cohort was higher among hospitalised cases than among those not hospitalised (54% vs 46%), slightly lower in the age group above 65 years compared to other age groups (41 % vs 46–47%) and slightly lower in cases with more than 2 comorbidities (43% vs 46–47%). The differences in county of residence, sampling week, age, number of comorbidities were considered minor for our study design and aim. Regarding the 8% difference between those hospitalised and those not, we conducted a sensitivity analysis which concluded that this did not influence our estimates that are presented in the published manuscript (see 3.2).

In table S3, we also present the number of hospitalisations among all notified cases. The proportion of cases hospitalised among notified cases increased with age, number of comorbidities and was higher among men and persons born outside of Norway. The findings were similar to the ones for cases in our study cohort. The proportions are slightly lower here than the ones reported among our study cohort (see table 1 in the manuscript) which is expected since the proportion of cases in our study cohort among hospitalised was slightly higher.

**Table S3: Characteristics of notified cases and cases in our study cohort (B.1.1.7, B.1.351 and non-VOC), Norway, 28 December 2020 – 2 May 2021**

| **Characteristics** | | **All notified cases (%)** | **Study cohort** | | **Hospitalised cases** | |
| --- | --- | --- | --- | --- | --- | --- |
|  |  |  | **n** | **% of all notified** | **n** | **% of all notified** |
| Total | | 60,606 (100 %) | 28,301 | 47 % | 1,901 | 3.1 % |
| Sex | Female | 28,527 (47 %) | 13,292 | 47 % | 746 | 2.6 % |
|  | Male | 32,079 (53 %) | 15,009 | 47 % | 1,155 | 3.6 % |
|  |  |  | P=0.634 | | P<0.0001 | |
| Age group | 0-24 years | 24,826 (41 %) | 11,782 | 47 % | 73 | 0.3 % |
|  | 25-44 years | 19,973 (33 %) | 9,370 | 47 % | 435 | 2.2 % |
|  | 45-64 years | 13,257 (22 %) | 6,103 | 46 % | 894 | 6.7 % |
|  | ≥65 years | 2,550 (4.2 %) | 1,046 | 41 % | 499 | 20 % |
|  |  |  | P<0.0001 | | P<0.0001 | |
| Norwegian born | Yes | 38,443 (63 %) | 17,865 | 46 % | 871 | 2.3 % |
|  | No | 21,533 (36 %) | 10,186 | 47 % | 968 | 4.5 % |
|  | Unknown | 630 (1.0 %) | 250 | 40 % | 62 | 9.8 % |
|  |  |  | P<0.0001 | | P<0.0001 | |
| Number of comorbidities | 0 | 53,064 (88 %) | 24,863 | 47 % | 1,098 | 2.1 % |
|  | 1 | 6,372 (11 %) | 2,940 | 46 % | 541 | 8.5 % |
|  | ≥2 | 1,170 (1.9 %) | 498 | 43 % | 262 | 22 % |
|  |  |  | P=0.009 | | P<0.0001 | |
| Period of diagnosis | Weeks 53-1 | 7,781 (13 %) | 635 | 8.2 % | 196 | 2.5 % |
|  | Weeks 2-3 | 4,471 (7.4 %) | 824 | 18 % | 118 | 2.6 % |
|  | Weeks 4-5 | 3,369 (5.6 %) | 1,680 | 50 % | 78 | 2.3 % |
|  | Weeks 6-7 | 3,466 (5.7 %) | 2,225 | 64 % | 107 | 3.1 % |
|  | Weeks 8-9 | 6,812 (11 %) | 5,069 | 74 % | 249 | 3.7 % |
|  | Week 10-11 | 11,548 (19 %) | 5,049 | 44 % | 422 | 3.7 % |
|  | Weeks 12-13 | 10,170 (17 %) | 4,358 | 43 % | 346 | 3.4 % |
|  | Weeks 14-15 | 7,696 (13 %) | 4,651 | 60 % | 265 | 3.4 % |
|  | Week 16-17 | 5,293 (8.7 %) | 3,810 | 72 % | 120 | 2.3 % |
|  |  |  | P<0.0001 | | P<0.0001 | |
| County of residence | Agder | 2,042 (3.4 %) | 1,148 | 56 % | 49 | 2.4 % |
|  | Innlandet | 1,863 (3.1 %) | 836 | 45 % | 72 | 3.9 % |
|  | Møre and Romsdal | 786 (1.3 %) | 283 | 36 % | 33 | 4.2 % |
|  | Nordland | 740 (1.2 %) | 476 | 64 % | 27 | 3.7 % |
|  | Oslo | 19,028 (31 %) | 9,827 | 52 % | 622 | 3.3 % |
|  | Rogaland | 3,661 (6.0 %) | 1,668 | 46 % | 135 | 3.7 % |
|  | Troms and Finnmark | 505 (0.8 %) | 156 | 31 % | 14 | 2.8 % |
|  | Trøndelag | 1,720 (2.8 %) | 324 | 19 % | 31 | 1.8 % |
|  | Vestfold and Telemark | 3,999 (6.6 %) | 1,641 | 41 % | 109 | 2.7 % |
|  | Vestland | 3,168 (5.2 %) | 1,812 | 57 % | 91 | 2.9 % |
|  | Viken | 23,054 (38 %) | 10,107 | 44 % | 716 | 3.1 % |
|  | Unknown | 40 (0.1 %) | 23 | 58 % | 2 | 5.0 % |
|  |  |  | P<0.0001 | |  | P=0.002 |
| Hospitalised | Yes | 1,901 (3.1 %) | 1,017 | 54 % | NA | NA |
|  | No | 58,705 (97 %) | 27,284 | 46 % | NA | NA |
|  |  |  | P<0.0001 | |  | NA |

Note: p-values presented are from chi-square tests.

### 3.2 Sensitivity analyses

In addition to the main analysis, we also conducted a number of sensitivity analyses to further explore our results regarding the risk of hospitalisation. The estimates from our sensitivity analyses are presented in table S4. The methodology used was the same as the one used in the main analysis (see table 2 in the manuscript). We modified the study population, by including or excluding some of the cases in our study cohort, and definition of hospitalisation. To take into account the observed differences in our study cohort between hospitalised and non-hospitalised cases, we reanalysed the main data by adding weights to a logistic regression model. The results of this analysis were aOR 2.34 (95%CI 2.15–2.55) for B.1.1.7, and aOR 2.44 (95%CI 1.97–3.02) for B.1.351, which were consistent with the main analysis (table S4).

**Table S4. Infection with variant B.1.1.7, B.1.351 and risk or odds of hospitalisation compared to non-VOC when modifying the study population or definition of outcome, Norway, 28 December 2020 – 2 May 2021**

| **Analysis** | **Non-VOC cases** | **B.1.1.7 cases** | | **B.1.351 cases** | |
| --- | --- | --- | --- | --- | --- |
|  | **Hospitalised cases (%)** | **Hospitalised cases (%)** | **Adjusted RR* (95% CI)** | **Hospitalised cases (%)** | **Adjusted RR* (95% CI)** |
| Main analysis (n=28,301) | 110 (2.4) | 884 (3.8) | 1.95 (1.61-2.35) | 23 (4.2) | 2.37 (1.71-3.27) |
| Main analysis when using logistic regression and calculated aOR** instead of aRR* (n=28,301) | 110 (2.4) | 884 (3.8) | 2.20 (1.78-2.72) | 23 (4.2) | 2.67 (1.62-4.38) |
| Changes in the study population or definition of outcome |  | | | | |
| Including as hospitalised those that were hospitalised with COVID-19 (as the main or secondary cause) 2 days before sampling or 14 days after sampling & had duration of hospitalisation >12 hours (n=28,481)*** | 123 (2.7 %) | 977 (4.2 %) | 1.92 (1.61-2.29) | 24 (4.4 %) | 2.22 (1.64-3.01) |
| Including as hospitalised 180 cases in our study cohort that had COVID-19 as secondary cause of hospitalization (n=28,481) | 142 (3.1 %) | 1,031 (4.4 %) | 1.75 (1.49-2.07) | 24 (4.4 %) | 2.09 (1.58-2.77) |
| Including as non-hospitalised 180 cases in our study cohort that had COVID-19 as secondary cause of hospitalization (n=28,481) | 110 (2.4 %) | 884 (3.8 %) | 1.94 (1.61-2.35) | 23 (4.2 %) | 2.39 (1.63-3.49) |
| Excluding 80 cases that had duration of hospitalisation ≤1 day (n=28,221) | 98 (2.1) | 817 (3.5 %) | 2.04 (1.67-2.49) | 22 (4.0 %) | 2.50 (1.78-3.50) |
| Including 1,037 cases in our study cohort that were vaccinated with at least one eligible dose (n=29,338) | 114 (2.4 %) | 944 (3.9 %) | 1.90 (1.58-2.28) | 27 (4.6 %) | 2.14 (1.50-3.08) |
| Excluding 15,554 cases in our study cohort that were not sequenced (only had PCR result) (n=12,747) | 84 (2.7 %) | 406 (4.4 %) | 2.01 (1.61-2.51) | 21 (4.7 %) | 2.34 (1.65-3.31) |
| When limiting the analysis from week 7 to week 17 (Mid Feb-May) when the testing strategy in Norway was enhanced and stable (n=24,242) | 36 (2.1 %) | 829 (3.8 %) | 2.04 (1.49-2.80) | 21 (4.5 %) | 2.66 (1.72-4.12) |

* aRR: adjusted relative risk; CI: confidence intervals. Adjusted for sex, age group, country of birth and number of comorbidities (as in table 2 in the manuscript).

** aOR: adjusted odds ratio.

*** Similar to the definition of hospitalisation used in the preprint publication from Denmark in (4).

### 3.3 Results from logistic regression when the variant was included as a 3-level categorical variable

Here we present the results from the univariate and multivariable logistic regression adjusted for variant (categorical variable with three levels in this model), sex, age group, country of birth and number of underlying comorbidities. Logistic regression was used in this part of analysis since binomial regression did not converge. This analysis allows readers to see the estimates for hospitalisation for the rest of the variables that were included in the multivariable analysis and are not presented in our other analyses in the main results of the manuscript.

**Table S5. Odds ratios for hospitalisation, Norway, 28 December 2020 – 2 May 2021. Results from univariate and multivariable logistic regression adjusted for variant (categorical variable with three levels), sex, age group, country of birth and number of underlying comorbidities.**

|  |  | **Hospitalisation** | | | |  | |  |
| --- | --- | --- | --- | --- | --- | --- | --- | --- |
|  |  |  | **No** | **Yes (%)** | **Crude OR* (95% CI)** | | **Adjusted OR* (95% CI)** | |
| Variant | Non-VOC | | 4,474 | 110 (2.4) | Ref | | Ref | |
|  | B.1.1.7 variant | | 22,284 | 884 (3.8) | 1.61 (1.32-1.97) | | 2.20 (1.79-2.72) | |
|  | B.1.351 variant | | 525 | 23 (4.2) | 1.78 (1.13-2.81) | | 2.54 (1.56-3.11) | |
| Sex | Female | | 12,887 | 405 (3.1) | Ref | | Ref | |
|  | Male | | 14,397 | 612 (4.1) | 1.35 (1.19-1.54) | | 1.32 (1.15-1.50) | |
| Age group | 0-24 years | | 11,741 | 41 (0.4) | Ref | | Ref | |
|  | 25-44 years | | 9,134 | 236 (2.5) | 7.40 (5.30-10.32) | | 6.45 (4.60-9.03) | |
|  | 45-64 years | | 5,607 | 496 (8.1) | 25.33 (18.39-34.88) | | 19.22 (13.88-26.59) | |
|  | ≥65 years | | 802 | 244 (23) | 87.12 (62.11-122.22) | | 54.78 (38.54-77.87) | |
| Norwegian born | Yes | | 17,387 | 478 (2.7) | Ref | | Ref | |
|  | No | | 9,672 | 514 (5.1) | 1.93 (1.70-2.19) | | 1.50 (1.32-1.73) | |
|  | Unknown | | 225 | 25 (10) | 4.04 (2.65-6.17) | | 0.85 (0.54-1.34) | |
| Number of comorbidities | 0 | | 24,266 | 597 (2.4) | Ref | | Ref | |
|  | 1 | | 2,647 | 293 (10) | 4.50 (3.89-5.20) | | 2.50 (2.14-2.93) | |
|  | ≥2 | | 371 | 127 (26) | 13.91 (11.20-17.29) | | 4.37 (3.43-5.56) | |

* OR: odds ratio; CI: confidence intervals
