# Supplementary material for: Increased risk of hospitalisation and intensive care admission associated with reported cases of SARS-CoV-2 variants B.1.1.7 and B.1.351 in Norway, December 2020 –May 2021
Source: PLoS One. 2021 Oct 11;16(10):e0258513. doi: 10.1371/journal.pone.0258513 (PMC8504717; doi:10.1371/journal.pone.0258513)
Supplement: Attachment — Submitted filename: reviewer_responses_risk_hospitalisation_B117_B1351_Norway.docx [file pone.0258513.s002.docx]

**Authors responses to editors’ and reviewers’ comments for the manuscript PONE-D-21-21292.**

| **Editors** | **Authors response** |
| --- | --- |
| Please ensure that your manuscript meets PLOS ONE's style requirements, including those for file naming. The PLOS ONE style templates can be found at  <https://journals.plos.org/plosone/s/file?id=wjVg/PLOSOne_formatting_sample_main_body.pdf> and  <https://journals.plos.org/plosone/s/file?id=ba62/PLOSOne_formatting_sample_title_authors_affiliations.pdf> | *We have reformatted our manuscript, according to the style requirements in the links provided.* |
| Please provide additional details regarding participant consent. In the ethics statement in the Methods and online submission information, please ensure that you have specified (1) whether consent was informed and (2) what type you obtained (for instance, written or verbal, and if verbal, how it was documented and witnessed). If your study included minors, state whether you obtained consent from parents or guardians. If the need for consent was waived by the ethics committee, please include this information.  If you are reporting a retrospective study of medical records or archived samples, please ensure that you have discussed whether all data were fully anonymized before you accessed them and/or whether the IRB or ethics committee waived the requirement for informed consent. If patients provided informed written consent to have data from their medical records used in research, please include this information. Once you have amended this/these statement(s) in the Methods section of the manuscript, please add the same text to the “Ethics Statement” field of the submission form (via “Edit Submission”).  For additional information about PLOS ONE ethical requirements for human subjects research, please refer to <http://journals.plos.org/plosone/s/submission-guidelines#loc-human-subjects-research>. | *In lines 182 – 183 (in the revised manuscript with track changes) we have specified that the need for informed consent was waived by the ethics committee, in line with the Norwegian Health Research Act.* |
| We note that you have indicated that data from this study are available upon request. PLOS only allows data to be available upon request if there are legal or ethical restrictions on sharing data publicly. For information on unacceptable data access restrictions, please see <http://journals.plos.org/plosone/s/data-availability#loc-unacceptable-data-access-restrictions>.  In your revised cover letter, please address the following prompts:   a) If there are ethical or legal restrictions on sharing a de-identified data set, please explain them in detail (e.g., data contain potentially identifying or sensitive patient information) and who has imposed them (e.g., an ethics committee). Please also provide contact information for a data access committee, ethics committee, or other institutional body to which data requests may be sent.   b) If there are no restrictions, please upload the minimal anonymized data set necessary to replicate your study findings as either Supporting Information files or to a stable, public repository and provide us with the relevant URLs, DOIs, or accession numbers. Please see <http://www.bmj.com/content/340/bmj.c181.long> for guidelines on how to de-identify and prepare clinical data for publication. For a list of acceptable repositories, please see <http://journals.plos.org/plosone/s/data-availability#loc-recommended-repositories>.  We will update your Data Availability statement on your behalf to reflect the information you provide. | *The data analysed in this study come from the Norwegian* *national emergency preparedness register, Beredt C19. Beredt C19 was established under the Health Preparedness Act §2-4 so that the Norwegian Institute of Public Health (NIPH) can quickly obtain the necessary knowledge about the COVID-19 epidemic, enabling the authorities to assess risk and implement measures to safeguard the health of the entire population. Beredt C19 contains individual-level data from central health registries, national clinical registries and other national administrative registries.*  *In Beredt C19, only fully anonymised data (i.e. data that are neither directly nor potentially indirectly identifiable) are permitted to be taken out of the secure zone at the NIPH where the data are stored (and where the researchers had access to the data). This is in line with the approved DPIA for Beredt C19, which is grounded in the Health Preparedness Act. Legal restrictions therefore prevent us from sharing a dataset publicly that would enable others to replicate our study findings.*  *However, external researchers are freely able to request access to linked data from the different registries from outside the structure of Beredt C19. Further information on Beredt C19, including contact information for the Beredt C19 project manager, and information on access to data from each individual data source, is available at* [*https://www.fhi.no/en/id/infectious-diseases/coronavirus/emergency-preparedness-register-for-covid-19/*](https://www.fhi.no/en/id/infectious-diseases/coronavirus/emergency-preparedness-register-for-covid-19/)*.* |
| Please include captions for your Supporting Information files at the end of your manuscript, and update any in-text citations to match accordingly. Please see our Supporting Information guidelines for more information: <http://journals.plos.org/plosone/s/supporting-information>. | *In lines 366 – 368 we have added a caption for the supplementary materials (S1) and updated the in-text citations.* |
| **Reviewer 1** | **Authors response** |
| **Introduction** |  |
| In the introduction section, the authors should update the current status of SARS-CoV-2 variants, especially the delta variant. | *In lines 81 – 82 we have updated our description of the dominant circulating strains in Norway, including that B.1.617.2 (delta variant) began to predominate from week 27 2021.* |
| What is the policy of Norway health system while patients infected with SARS-CoV-2 was identified? | *In lines 87 – 88 we have specified that during the study period “the hospital system functioned within capacity, and hospital treatment was available to all patients who would benefit”.* |
| Several descriptions are not clear. For example, “The proportion of confirmed cases of COVID-19 with known virus variant…”, this description is unclear and should be specified; “…. the dominating variant since week 7, 2021”, what is the proportion of patients infected with this variant and the data source? | *In lines 75 – 77 we have specified more clearly that “The proportion of all laboratory-confirmed cases of COVID-19 in Norway who had available data on the variant of SARS-CoV-2 that caused their infection….”*  *In line 75 we have added the data source for the data on virus variants. In lines 79 – 82 we have specified that “B.1.1.7 was the variant detected in at least 50% of cases from week 5 (including over 90% weekly from week 13 to week 20), until week 27 2021, when the VOC B.1.617.2 (delta variant) began to predominate.”.* |
| **Results** |  |
| The resolution of figure 1 is poor | *Thanks for your comment. We have resubmitted figure 1 as a tif file, as per the journal style requirements.* |
| From line 196 to line 202, it is unclear about “the proportion of cases in our study….” and “For the higher proportion of cases in our study…”, please makes them clearer | *We have revised lines 209 – 225 to make them clearer to the reader.* |
| As the authors described, “the proportion of cases with known variant data increased from 8% in weeks 53/2020–1/2021 to 78% in weeks 8–9 and stayed between 46 to 75% 181 during weeks 10–17”, there is a wide variation in the proportion of patients receiving variant testing. For those cases not receiving testing, especially in the earlier weeks of this study, how many cases were admitted to hospital or ICU? | *Many thanks for your comment. Data on hospitalisation for those with known variant compared to all cases is given in S1 table S3. Of those with unknown variant (32 305 cases) 884 (2.7%) were admitted to hospital. As detailed in the manuscript, among all hospitalised cases, 54% were included in our study cohort, while among non-hospitalised cases 46% were included. We conducted a sensitivity analysis which indicated that the 8% difference between those hospitalised and not hospitalised did not influence our estimates in our main analysis as presented below (S1, part 3.2).* |
| **General comments** |  |
| Finally, two main problems of this study should be clarified. First, how about the disease severity of included patients at presentation. Second, how many days have been passed since the onset of symptoms among the included patients at presentation. | *We do not have data on severity of disease at presentation, however, in lines 87 – 89 we have specified that during the study period “the hospital system functioned within capacity, and hospital treatment was available to all patients who would benefit”.*  *In lines 242 – 247 we have added data on the median time from symptom onset to hospitalisation in our study cohort. In light of this, we have also adjusted lines 147 – 149 in the methods.* |
| **Reviewer 2** | **Authors response** |
| **General comments** |  |
| This article, entitled “Increased risk of hospitalisation and intensive care admission associated with infection with SARS-CoV-2 variants B.1.1.7 and B.1.351 in Norway, December 2020–May 2021”, described the increased severity of COVID-19 illnesses of VOC strains, B.1.1.7 and B.1.351. The data analysis is stringent and persuasive. | *Many thanks for your thorough review.* |
| **Abstract** |  |
| The authors may clarify the sentence ” Among hospitalised cases, no difference was found in the risk of ICU admission between B.1.1.7 and non-VOC”, which was not parallel with the title of the article. | *The results “with infection with SARS-CoV-2 variants” as referred to the title are in parallel, but we understand your point that some people may find it confusing. We have rephrased the title as “Increased risk of hospitalisation and intensive care admission associated with* ***reported cases*** *of SARS-CoV-2 variants B.1.1.7 and B.1.351 in Norway, December 2020 – May 2021”*  *If this is still considered unclear, another alternative would be to rephrase the title as “Risk of hospitalisation and intensive care admission associated with infection with SARS-CoV-2 variants B.1.1.7 and B.1.351 in Norway, December 2020 – May 2021”, i.e. dropping the word ‘increased’.* |
| **Methods** |  |
| According to the manuscript, the study population had excluded the persons without national identity number. Please describe the characteristics of COVID-19 confirmed cases who did not have national identity number, like immigrants, foreign workers, or visitors. Did these cases have different risks of hospitalization and intensive care admission? | *The national identity number was essential to link data from all registries used in the analysis. If we would have included cases without a national identity number, this would have added 625 cases to the final study cohort (n=28,926). Cases without a national identity number had a marginally lower crude risk of hospitalisation (RR with binary regression: 0.58, 95%CI: 0.34 – 0.99; OR with logistic regression: 0.57, 95%CI: 0.33 – 0.99), although this relationship was not sustained in the multivariable model (aOR 0.57, 95%CI 0.31 – 1.06). Logistic regression was used in the multivariable model, as the binary regression model did not converge. It is adjusted for age, gender, variant and country of birth, but not for underlying comorbidities, which are only available for cases with a national identity number. We did not observe a difference in the crude odds of ICU admission (RR from binary regression: 0.88, 95%CI: 0.33 – 2.35) for cases not registered with a national identity number, compared to those registered with a national identity number.* |
| **Results** |  |
| The authors had excluded the cases who had received COVID-19 vaccines. Do the authors think that vaccine is a protective factors? Did the authors do sensitivity analysis including the vaccinees? | *In table 3 of the WHO epidemiological report from 20 July 2021 (*[*https://www.who.int/publications/m/item/weekly-epidemiological-update-on-covid-19---20-july-2021*](https://www.who.int/publications/m/item/weekly-epidemiological-update-on-covid-19---20-july-2021)*), the most recent evidence of vaccine performance against VOC is summarised. For both B.1.1.7 and B.1.351, current evidence suggests that several vaccines reduce the risk of severe disease among those infected with these variants, although for some vaccines the level of protection is reduced (e.g. Astra Zeneca for B.1.1.7).*  *In S1, part 3.2 we present the results from a sensitivity analysis* *including the vaccinees.* |
| Please make a correction on the figure legend: Figure 1. Distribution of COVID-19 cases reported and screened with B.1.1.7, B.1.351 and non-VOC by week of sampling, Norway, December 2020 – May 2021. Note: ' All cases reported' (n=60,606) excludes vaccinated reported cases, cases who had a national identity number registered and…. | *In line 196 we have corrected this to “…cases who* ***did not have*** *a national identity number registered…”* |
| May the authors explain the meanings of the sentences at line 196: The proportion of cases in our study cohort was higher among hospitalised cases than among those not hospitalised (54% vs 46%)…... | *We have revised lines 209 – 225 to make them clearer to the reader.* |
| Table 2 showed that the aRR was highest among patients infected with B.1.1.7 without comorbidity, compared with non-VOC. Please explain. | *The aim of the stratified analysis in table 2 was to show that our results were robust in most of the strata. In the stratification by comorbidities, our CI are overlapping within the different strata but still suggesting a higher risk of hospitalisation in cases with alpha vs non-VOC. The higher RR estimate in the strata with no comorbidities does not mean that there is any interaction with this variable. We have checked for interactions (line 156 – 157) and none were found.* |
| **Discussion** |  |
| Regarding the variants B.1.1.7 and B.1.351, increased rates of hospitalization and ICU admission had been described in Denmark, England, South Africa, and European pooled data. Did the authors consider what the uniqueness of this article? | *It is correct that other countries have published estimates of the risk of severe disease regarding B.1.1.7 and/or B.1.351. Given the spread of VOC and impact of the COVID-19 pandemic worldwide, timely analysis on the association between current and future VOC, and the risk of severe disease remains essential to ensure societies and health systems are best prepared and able to appropriately respond to this evolving public health threat. This requires studies from a variety of settings, considering local epidemiological characteristics, to ensure an in-depth understanding of changes in severity of disease related to different variants of SARS-CoV-2.*  *A significant strength of our study is that we have analysed individual-level data on a representative sample of cases in Norway, controlling for a range of potential confounders. To our knowledge, no other study has reported estimates on a representative sample of cases for both B.1.1.7 and B.1.351. The pooled EU study analysed a cohort that represented <1% of all cases in the included countries in the study period. They also analysed routine surveillance data which have been limited by underreporting of outcomes. Also, as far as we are aware, the published evidence for B.1.351 in particular remains scarce. Aside from the pooled EU study, we are only aware of studies from South Africa, which do not analyse individual-level variant data, and do not report on the risk for hospitalisation or ICU admission. According to the WHO epidemiological report from 20 July 2021 (*[*https://www.who.int/publications/m/item/weekly-epidemiological-update-on-covid-19---20-july-2021*](https://www.who.int/publications/m/item/weekly-epidemiological-update-on-covid-19---20-july-2021)*) the association between B.1.351 and disease severity is “Not confirmed”, and therefore more studies on B.1.351 in particular are essential.* |
| The conclusion of this article “There is a need for further studies on differences in patient trajectories and clinical characteristics among patients infected with VOC in hospitalised cohorts” was not relevant and did not echo with the discussions. The authors may conclude with preparedness for the increasing hospitalization and ICU admission. | *We have deleted the final sentence in the discussion (line 363 – 364).* |
| **Other comments from Authors** |  |
| *We have updated reference 13, which has now been published in a peer-reviewed journal. In our original submission, this reference was to a preprint version. We have also added reference 17 and 27, and updated the related text in the discussion accordingly.* | |
| Date Received: | *19-Jul-2021* |
| Date Responded: | *26-Jul-2021* |
