# Supplementary material for: Increased risk of hospitalisation and intensive care admission associated with reported cases of SARS-CoV-2 variants B.1.1.7 and B.1.351 in Norway, December 2020 –May 2021
Source: PLoS One. 2021 Oct 11;16(10):e0258513. doi: 10.1371/journal.pone.0258513 (PMC8504717; doi:10.1371/journal.pone.0258513)
Supplement: Attachment — Submitted filename: reviewer_responses_risk_hospitalisation_B117_B1351_Norway.docx [file pone.0258513.s003.docx]

**Authors responses to editors’ and reviewers’ comments for the manuscript** **PONE-D-21-21292R1.**

| **Editors** | **Authors response** |
| --- | --- |
| Please review your reference list to ensure that it is complete and correct. If you have cited papers that have been retracted, please include the rationale for doing so in the manuscript text, or remove these references and replace them with relevant current references. Any changes to the reference list should be mentioned in the rebuttal letter that accompanies your revised manuscript. If you need to cite a retracted article, indicate the article’s retracted status in the References list and also include a citation and full reference for the retraction notice. | *We have revised our reference list, as per the PLOS One author guidelines:* [*https://journals.plos.org/plosone/s/submission-guidelines#loc-references*](https://journals.plos.org/plosone/s/submission-guidelines#loc-references) |
| **Reviewer 1** | **Authors response** |
| Regarding the policy of government health system, are all patients infected with SARS-CoV-2 admitted or only those with conditional criteria, such as moderate to severe severity, hospitalized? During the study period, were the standards of hospitalization for patients with SARS-CoV-2 consistent? | *Patients are only admitted to hospital in Norway if they would benefit from hospital treatment, that’s to say that they are severely ill enough to require hospital treatment. The standards of hospitalisation were consistent during the study period. As mentioned in line 78–80 “During this period the hospital system functioned within capacity, and hospital treatment was available to all patients who would benefit”. We have added “**Hospital admission criteria were consistent.” in line 80, to further clarify this statement.* |
| How about the comparison of the median time from symptom onset to hospitalization between various group? Data about this parameter and others (testing time to hospitalization) between groups should be presented in the article. Over half of patients had no records about the time from symptom onset to hospitalization (line 242 -243). How about the proportion of the missing data among different groups? | *Many thanks for your comment. The aim of this study is to investigate the relative risk of hospital and ICU admission associated with different variants of SARS-CoV-2 in Norway. Our study was conducted in a period when the hospital system functioned within capacity and standards for hospitalisation did not change.*  *A comprehensive comparison of parameters related to patient trajectories would be interesting, however one that we believe is beyond the scope of this specific article. The most important comparison with regards to time from symptom onset or sampling to hospitalisation in the context of this analysis is between the variants. This is presented in line 222–226, and no statistical difference was observed between B.1.1.7, B.1.351 and non-VOC for these parameters. In our supplementary materials we have also included a range of sensitivity analyses, where we adjust our definition for who is considered as ‘hospitalised’ in our analysis, for example by length of stay and time from sampling date to admission. In these sensitivity analyses our results were robust. In addition, a more detailed multivariable analysis of the time from symptom onset to hospitalisation, and length of stay in hospital between B.1.1.7 and non-VOC has also been published (see reference 17). Again, no statistical difference was observed. Differences in parameters related to patient trajectories may of course exist between other groups, for example by age or underlying comorbidity, which are controlled for in our multivariable models.* |
| The decision of hospitalization may depend on objective parameters (disease severity, not available in this study) or subjective parameters (physician personal decision or the policy of healthcare system). If the decision of hospitalization is based on subjective parameter, selection bias may be present. Therefore, it is necessary to clarify the standards of hospitalization. | *Many thanks for your comment. Standards of hospitalisation were consistent during the study period, however subjective parameters are indeed a potential source of bias. We have already commented on this in our discussion, line 321–322 “Conversely, the increased concern regarding VOC could lead to clinicians hospitalising patients more often, which would cause an overestimation of the relevant RRs.”. We have expanded on this point by adding the sentence “As we did not have access to data on disease severity at admission, we cannot rule out this bias in our analysis.” in line 322–323.*  *In our supplementary materials we have included a range of sensitivity analyses, where we adjust our definition for who is considered as ‘hospitalised’ in our analysis, for example by main cause of hospitalisation, length of stay and time from sampling date to admission. In these sensitivity analyses our results were robust.* |
| The English grammar should be edited. | *We have revised our manuscript, and made grammatical corrections, where necessary.* |
| **Reviewer 2** | **Authors response** |
| The authors replied to the comments comprehensively, and had done very detail. I have no more comments. | *Many thanks for your time and effort in reviewing our manuscript. We are happy to hear that you are happy with our responses to your previous comments.* |
| **Additional authors comments** | |
| We have made some minor corrections to the text. For example, in line 67 ‘United Kingdom’ was deleted, as this abbreviation was already defined in lines 58–59. Also, we changed ‘relative risks’ to ‘risk ratios’ in line 141 to ensure that we used the same terminology throughout the manuscript. All such edits are tracked. | |
| Date Received: | *19-Aug-2021* |
| Date Responded: | *19-Aug-2021* |
